# Supplementary material for: Profiling of microglia nodules in multiple sclerosis reveals propensity for lesion formation
Source: Nat Commun. 2024 Feb 23;15:1667. doi: 10.1038/s41467-024-46068-3 (PMC10891081; doi:10.1038/s41467-024-46068-3)
Supplement: Supplementary file 1 — Supplementary Information [file 41467_2024_46068_MOESM1_ESM.pdf]

# Supplemental files

# Profiling of microglia nodules in multiple sclerosis reveals propensity for lesion formation

Aletta M.R. van den Bosch,[1] Marlijn van der Poel,[1] Nina L. Fransen,[1] Maria C.J. Vincenten,[1] Anneleen M. Bobeldijk,[1] Aldo Jongejan,[2] Hendrik J. Engelenburg,[1] Perry D. Moerland,[2] Joost Smolders,[1,3] Inge Huitinga,[1,4x] and Jörg Hamann[1,5x]

[1]Neuroimmunology Research Group, Netherlands Institute for Neuroscience, Amsterdam, The Netherlands; [2]Department of Epidemiology and Data Science, Amsterdam Public Health Research Institute, Amsterdam University Medical Center, Amsterdam, The Netherlands; [3]MS Center ErasMS, Departments of Neurology and Immunology, Erasmus Medical Center, Rotterdam, The Netherlands; [4]Swammerdam Institute for Life Sciences, University of Amsterdam, Amsterdam, The Netherlands; and [5]Department of Experimental Immunology, Amsterdam Institute for Infection and Immunity, Amsterdam University Medical Center, Amsterdam, The Netherlands.

[x]contributed equally

**Supplementary table 1: Outliers removed from RNA sequencing analysis**

| Sequence ID | Diagnosis | Tissue | Age | Sex |
|---|---|---|---|---|
| s104343_001_003 94-116 | Stroke | Microglia nodules | 72 | F |
| s104343_001_004 94-116 | Stroke | NAWM | 72 | F |
| s104343_001_028 17-100 | MS | NAWM | 66 | M |

**Supplementary table 2: Primers used for RT-qPCR on nodule tissue**

| Gene name | Forward sequence (5'-3') | Reverse sequence (5'-3') |
|---|---|---|
| EEF1A1 | AAGCTGGAAGATGGCCCTAAA | AAGCGACCCAAAGGTGGAT |
| GAPDH | TGCACCACCAACTGCTTAGC | GGCATGGACTGTGGTCATGA |
| IGKC | CCATCTGTCTTCATCTTCCCG | ATCCACCTTCCACTGTACTTTG |
| IGHG1 | GGTCAAAGGCTTCTATCCCAG | AGGCGTGGTCTTGTAGTTG |
| IGHG2 | GGAGATGACCAAGAACCAGG | GGAGGTGTGGTCTTGTAGTTG |
| IGKV3-15 | CCTCATCTATGGTGCATCCAC | TGCTGATGGTGAGAGTGAAC |

**Supplementary table 3: Top 50 upregulated DE genes in MS nodules vs MS nnNAWM.** Differential expression was assessed using an empirical Bayes moderated t-test within limma's linear model framework including the precision weights estimated by voom and the consensus correlation between samples of the same donor. The differential expression analysis was performed both with and without a covariate for the estimated microglia content (in percent). Resulting p values were corrected for multiple testing using the Benjamini-Hochberg false discovery rate.

| Gene symbol | Gene name | logFC | P. Adj. | LogFC cor. | P. Adj. Cor. |
|---|---|---|---|---|---|
| RGS16 | regulator of G protein signaling 16 | 10.186 | 2.27e-5 | 9.149 | 0.015 |
| CXCL16 | C-X-C motif chemokine ligand 16 | 10.074 | 2.11e-6 | 9.666 | 0.004 |
| TNFRSF1B | TNF receptor superfamily member 1B | 9.578 | 3.36e-6 | 9.079 | 0.006 |
| C1QA | complement C1q A chain | 9.451 | 1.40e-5 | 8.344 | 0.022 |
| CTSC | cathepsin C | 9.025 | 1.40e-5 | 7.611 | 0.033 |
| MX1 | MX dynamin like GTPase 1 | 8.935 | 1.40e-5 | 8.283 | 0.022 |
| RGS1 | regulator of G protein signaling 1 | 8.869 | 0.001 | 7.142 | 0.162 |
| EML4-AS1 | EML4 antisense RNA 1 | 8.846 | 1.21e-5 | 8.333 | 0.015 |
| IL18 | interleukin 18 | 8.773 | 1.21e-5 | 8.771 | 0.008 |
| NCKAP1L | NCK associated protein 1 like | 8.491 | 1.46e-5 | 7.309 | 0.033 |
| SRP68 | signal recognition particle 68 | 8.479 | 0.001 | 9.201 | 0.026 |
| SLC7A5 | solute carrier family 7 member 5 | 8.454 | 0.002 | 9.215 | 0.026 |
| LCP1 | lymphocyte cytosolic protein 1 | 8.352 | 0.001 | 7.297 | 0.162 |
| APBB1IP | amyloid beta precursor protein binding family B member 1 interacting protein | 8.350 | 0.003 | 7.614 | 0.147 |
| C1QB | complement C1q B chain | 8.315 | 0.001 | 7.831 | 0.037 |
| ERCC5 | ERCC excision repair 5, endonuclease | 8.267 | 1.85e-4 | 8.427 | 0.026 |
| SLCO2B1 | solute carrier organic anion transporter family member 2B1 | 8.257 | 0.002 | 7.878 | 0.051 |
| IGSF6 | immunoglobulin superfamily member 6 | 8.171 | 0.001 | 7.135 | 0.111 |
| CD84 | CD84 molecule | 8.169 | 0.001 | 5.701 | 0.342 |
| WARS1 | tryptophanyl-tRNA synthetase 1 | 8.121 | 0.002 | 7.574 | 0.156 |
| VAMP8 | vesicle associated membrane protein 8 | 8.111 | 0.001 | 6.343 | 0.270 |
| XAF1 | XIAP associated factor 1 | 8.090 | 0.001 | 7.407 | 0.156 |
| SAT2 | spermidine/spermine N1-acetyltransferase family member 2 | 8.049 | 0.006 | 8.251 | 0.153 |
| SCIN | scinderin | 7.971 | 0.019 | 6.159 | 0.402 |
| GPRIN3 | GPRIN family member 3 | 7.928 | 0.001 | 7.268 | 0.147 |
| MED11 | mediator complex subunit 11 | 7.922 | 0.001 | 7.171 | 0.170 |

| | | | | | |
|---|---|---|---|---|---|
| LPL | lipoprotein lipase | 7.916 | 0.002 | 5.355 | 0.395 |
| FCER1G | Fc fragment of IgE receptor Ig | 7.897 | 0.013 | 6.426 | 0.347 |
| CAMKK2 | calcium/calmodulin dependent protein kinase kinase 2 | 7.877 | 0.002 | 6.950 | 0.200 |
| RPGR | retinitis pigmentosa GTPase regulator | 7.790 | 1.85e-4 | 7.929 | 0.029 |
| ARHGAP24 | Rho GTPase activating protein 24 | 7.787 | 0.002 | 6.099 | 0.321 |
| ARHGAP18 | Rho GTPase activating protein 18 | 7.783 | 0.001 | 6.861 | 0.147 |
| MRPL55 | mitochondrial ribosomal protein L55 | 7.750 | 0.011 | 6.202 | 0.453 |
| HLA-DPB1 | major histocompatibility complex, class II, DP beta 1 | 7.735 | 0.008 | 7.106 | 0.203 |
| PLEK | pleckstrin | 7.697 | 0.003 | 5.824 | 0.321 |
| CD14 | CD14 molecule | 7.690 | 0.002 | 6.245 | 0.231 |
| LPAR6 | lysophosphatidic acid receptor 6 | 7.646 | 0.001 | 7.031 | 0.051 |
| EML4 | EMAP like 4 | 7.638 | 0.004 | 7.184 | 0.147 |
| TRIM52 | tripartite motif containing 52 | 7.613 | 0.002 | 8.153 | 0.070 |
| SERPINA1 | serpin family A member 1 | 7.551 | 0.006 | 6.582 | 0.353 |
| SLC2A5 | solute carrier family 2 member 5 | 7.542 | 0.002 | 5.592 | 0.373 |
| MPPE1 | metallophosphoesterase 1 | 7.492 | 0.002 | 7.091 | 0.164 |
| ETV5 | ETS variant transcription factor 5 | 7.488 | 0.003 | 7.152 | 0.188 |
| TANC1 | tetratricopeptide repeat, ankyrin repeat and coiled-coil containing 1 | 7.484 | 0.009 | 7.899 | 0.093 |
| DAGLB | diacylglycerol lipase beta | 7.432 | 0.001 | 7.622 | 0.053 |
| ITGAX | integrin subunit alpha X | 7.412 | 0.006 | 6.118 | 0.352 |
| LAPTM5 | lysosomal protein transmembrane 5 | 7.410 | 0.029 | 6.363 | 0.334 |
| RCOR1 | REST corepressor 1 | 7.404 | 0.006 | 5.679 | 0.407 |
| AXL | AXL receptor tyrosine kinase | 7.396 | 0.003 | 6.047 | 0.342 |

**Supplementary table 4: Upregulated DE genes in stroke nodules vs stroke nnNAWM.** Differential expression was assessed using an empirical Bayes moderated t-test within limma's linear model framework including the precision weights estimated by voom and the consensus correlation between samples of the same donor. The differential expression analysis was performed both with and without a covariate for the estimated microglia content (in percent). Resulting p values were corrected for multiple testing using the Benjamini-Hochberg false discovery rate.

| Gene symbol | description | logFC | P. Adj. | LogFC cor. | P. Adj. Cor. |
|---|---|---|---|---|---|
| BICD2 | BICD cargo adaptor 2 | 10.061 | 1.97e-5 | 9.803 | 2.85e-4 |
| NEB | nebulin | 9.979 | 3.37e-5 | 9.785 | 4.50e-4 |
| HLA-B | major histocompatibility complex, class I, B | 9.439 | 0.005 | 9.061 | 0.010 |
| C1QB | complement C1q B chain | 9.042 | 0.001 | 8.891 | 0.002 |
| APOC1 | apolipoprotein C1 | 8.731 | 0.005 | 8.497 | 0.009 |
| SLC11A1 | solute carrier family 11 member 1 | 8.227 | 0.005 | 7.755 | 0.010 |
| ASCC3 | activating signal cointegrator 1 complex subunit 3 | 7.989 | 0.006 | 8.115 | 0.013 |
| DIS3L2 | DIS3 like 3'-5' exoribonuclease 2 | 7.840 | 0.044 | 8.301 | 0.072 |
| WASL | WASP like actin nucleation promoting factor | 7.779 | 0.018 | 7.885 | 0.033 |
| RPGR | retinitis pigmentosa GTPase regulator | 6.599 | 0.037 | 6.644 | 0.073 |

**Supplementary table 5: Top 50 upregulated DE genes in MS nodules vs stroke nodules.** Differential expression was assessed using an empirical Bayes moderated t-test within limma's linear model framework including the precision weights estimated by voom and the consensus correlation between samples of the same donor. The differential expression analysis was performed both with and without a covariate for the estimated microglia content (in percent). Resulting p values were corrected for multiple testing using the Benjamini-Hochberg false discovery rate.

| Gene symbol | Gene name | logFC | P. Adj. | LogFC cor. | P. Adj. Cor. |
|---|---|---|---|---|---|
| MRPS34 | mitochondrial ribosomal protein S34 | 8.639 | 0.002 | 8.948 | 0.031 |
| ABCF3 | ATP binding cassette subfamily F member 3 | 8.317 | 0.002 | 8.480 | 0.034 |
| GTPBP6 | GTP binding protein 6 (putative) | 8.294 | 0.004 | 8.396 | 0.055 |
| SEPTIN4-AS1 | SEPTIN4 antisense RNA 1 | 8.016 | 0.002 | 7.928 | 0.031 |
| KIN | Kin17 DNA and RNA binding protein | 7.841 | 0.004 | 8.281 | 0.056 |
| CYB5B | cytochrome b5 type B | 7.780 | 0.004 | 7.880 | 0.056 |
| CNPY3 | canopy FGF signaling regulator 3 | 7.730 | 0.005 | 7.352 | 0.068 |
| MFN2 | mitofusin 2 | 7.693 | 0.010 | 7.812 | 0.068 |
| NBR1 | NBR1 autophagy cargo receptor | 7.668 | 0.003 | 7.710 | 0.031 |
| HLA-DRB5 | major histocompatibility complex, class II, DR beta 5 | 7.643 | 0.002 | 7.632 | 0.037 |
| COPG1 | COPI coat complex subunit gamma 1 | 7.614 | 0.004 | 7.119 | 0.080 |
| FABP5 | fatty acid binding protein 5 | 7.601 | 0.002 | 6.942 | 0.056 |
| ACBD5 | acyl-CoA binding domain containing 5 | 7.540 | 0.004 | 7.955 | 0.055 |
| TMEM147 | transmembrane protein 147 | 7.528 | 0.013 | 7.198 | 0.110 |
| FXYD5 | FXYD domain containing ion transport regulator 5 | 7.504 | 0.004 | 7.183 | 0.068 |
| CHMP1A | charged multivesicular body protein 1A | 7.495 | 0.006 | 7.510 | 0.056 |
| ISG15 | ISG15 ubiquitin like modifier | 7.460 | 0.004 | 6.667 | 0.098 |
| TRMT6 | tRNA methyltransferase 6 | 7.428 | 0.005 | 7.560 | 0.068 |
| UFC1 | ubiquitin-fold modifier conjugating enzyme 1 | 7.396 | 0.015 | 7.066 | 0.080 |
| HYI | hydroxypyruvate isomerase (putative) | 7.341 | 0.005 | 7.286 | 0.078 |
| PARP9 | poly(ADP-ribose) polymerase family member 9 | 7.279 | 0.013 | 7.503 | 0.080 |
| TRAPPC4 | trafficking protein particle complex 4 | 7.252 | 0.004 | 6.605 | 0.090 |
| SNX24 | sorting nexin 24 | 7.245 | 0.015 | 6.995 | 0.145 |
| RAB33A | RAB33A, member RAS oncogene family | 7.244 | 0.013 | 7.560 | 0.085 |
| PPME1 | protein phosphatase methylesterase 1 | 7.242 | 0.013 | 7.017 | 0.123 |
| TAX1BP1-AS1 | TAX1BP1 antisense RNA 1 | 7.226 | 0.004 | 7.011 | 0.068 |
| COMMD1 | copper metabolism domain containing 1 | 7.195 | 0.015 | 6.955 | 0.116 |

| | | | | | |
|---|---|---|---|---|---|
| UNC5C | unc-5 netrin receptor C | 7.194 | 0.016 | 7.622 | 0.080 |
| NOP16 | NOP16 nucleolar protein | 7.180 | 0.013 | 7.176 | 0.116 |
| H2AJ | H2A.J histone | 7.152 | 0.015 | 7.098 | 0.097 |
| APBB1IP | amyloid beta precursor protein binding family B member 1 interacting protein | 7.131 | 0.031 | 6.736 | 0.110 |
| DPF2 | double PHD fingers 2 | 7.131 | 0.013 | 7.098 | 0.120 |
| EXOC3 | exocyst complex component 3 | 7.121 | 0.014 | 6.854 | 0.123 |
| CHKA | choline kinase alpha | 7.084 | 0.008 | 7.747 | 0.055 |
| SS18 | SS18 subunit of BAF chromatin remodeling complex | 7.075 | 0.009 | 7.463 | 0.079 |
| IAH1 | isoamyl acetate hydrolyzing esterase 1 (putative) | 7.047 | 0.008 | 7.222 | 0.056 |
| UTP18 | UTP18 small subunit processome component | 7.029 | 0.008 | 6.589 | 0.118 |
| TXN | thioredoxin | 6.987 | 0.020 | 6.673 | 0.123 |
| TMEM222 | transmembrane protein 222 | 6.974 | 0.020 | 8.081 | 0.068 |
| ZFAND2B | zinc finger AN1-type containing 2B | 6.948 | 0.014 | 7.055 | 0.084 |
| PHACTR2 | phosphatase and actin regulator 2 | 6.940 | 0.013 | 6.893 | 0.120 |
| HIKESHI | heat shock protein nuclear import factor hikeshi | 6.924 | 0.008 | 7.451 | 0.068 |
| CENPO | centromere protein O | 6.910 | 0.005 | 6.822 | 0.078 |
| ARHGDIA | Rho GDP dissociation inhibitor alpha [Source:HGNC Symbol;Acc:HGNC:678] | 6.887 | 0.040 | 6.659 | 0.125 |
| UROD | uroporphyrinogen decarboxylase [Source:HGNC Symbol;Acc:HGNC:12591] | 6.878 | 0.010 | 6.396 | 0.123 |
| CLINT1 | clathrin interactor 1 [Source:HGNC Symbol;Acc:HGNC:23186] | 6.860 | 0.015 | 6.709 | 0.107 |
| ATP6V0E1 | ATPase H+ transporting V0 subunit e1 [Source:HGNC Symbol;Acc:HGNC:863] | 6.855 | 0.024 | 6.489 | 0.118 |
| EML4-AS1 | EML4 antisense RNA 1 [Source:HGNC Symbol;Acc:HGNC:40112] | 6.837 | 0.004 | 6.506 | 0.068 |
| SNX32 | sorting nexin 32 [Source:HGNC Symbol;Acc:HGNC:26423] | 6.831 | 0.026 | 6.178 | 0.209 |

**Supplementary table 6: Upregulated DE genes in MS nnNAWM vs stroke nnNAWM.** Differential expression was assessed using an empirical Bayes moderated t-test within limma's linear model framework including the precision weights estimated by voom and the consensus correlation between samples of the same donor. The differential expression analysis was performed both with and without a covariate for the estimated microglia content (in percent). Resulting p values were corrected for multiple testing using the Benjamini-Hochberg false discovery rate.

| Gene symbol | Gene name | logFC | P. Adj. | LogFC cor. | P. Adj. Cor. |
|---|---|---|---|---|---|
| RAD23A | RAD23 homolog A, nucleotide excision repair protein | 9.132 | 2.92e-5 | 9.139 | 3.78e-5 |
| NSUN5 | NOP2/Sun RNA methyltransferase 5 | 8.827 | 1.61e-5 | 8.825 | 2.75e-5 |
| COPA | COPI coat complex subunit alpha | 8.435 | 0.006 | 8.435 | 0.008 |
| NKAP | NFKB activating protein | 8.405 | 0.006 | 8.401 | 0.008 |
| ARFGAP2 | ADP ribosylation factor GTPase activating protein 2 | 8.202 | 0.006 | 8.219 | 0.008 |
| ATF7IP | activating transcription factor 7 interacting protein | 8.135 | 0.026 | 8.179 | 0.021 |
| GPAA1 | glycosylphosphatidylinositol anchor attachment 1 | 8.007 | 0.006 | 8.030 | 0.008 |
| KBTBD11 | kelch repeat and BTB domain containing 11 | 7.922 | 0.015 | 7.877 | 0.018 |
| HINT1 | histidine triad nucleotide binding protein 1 | 7.832 | 0.026 | 7.846 | 0.027 |
| ACO2 | aconitase 2 | 7.799 | 0.013 | 7.817 | 0.016 |
| EIF3G | eukaryotic translation initiation factor 3 subunit G | 7.759 | 0.010 | 7.764 | 0.012 |
| CPNE3 | copine 3 | 7.526 | 0.013 | 7.550 | 0.016 |
| ABL2 | ABL proto-oncogene 2, non-receptor tyrosine | 7.447 | 0.026 | 7.465 | 0.024 |
| ATP6V0E1 | ATPase H+ transporting V0 subunit e1 | 7.407 | 0.034 | 7.431 | 0.040 |
| SCNM1 | sodium channel modifier 1 | 7.364 | 0.046 | 7.398 | 0.045 |
| ARSB | arylsulfatase B | 7.217 | 0.013 | 7.243 | 0.016 |
| ARPC5 | actin related protein 2/3 complex subunit 5 | 7.180 | 0.046 | 7.234 | 0.049 |
| POLR3H | RNA polymerase III subunit H | 7.041 | 0.033 | 7.061 | 0.040 |
| CAPN3 | calpain 3 | 6.914 | 0.037 | 6.911 | 0.052 |
| SF3B6 | splicing factor 3b subunit 6 | 6.869 | 0.030 | 6.929 | 0.021 |
| STRN3 | striatin 3 | 6.729 | 0.047 | 6.755 | 0.060 |
| STAM2 | signal transducing adaptor molecule 2 | 6.554 | 0.047 | 6.573 | 0.060 |

**Supplementary table 7: DE genes of interest.** Genes of interest either found through literature or gene ontology analysis are shown for the various comparisons, sorted alphabetically. Differential expression was assessed using an empirical Bayes moderated t-test within limma's linear model framework including the precision weights estimated by voom and the consensus correlation between samples of the same donor. The differential expression analysis was performed both with and without a covariate for the estimated microglia content (in percent). Resulting p values were corrected for multiple testing using the Benjamini-Hochberg false discovery rate. LogFC and adjusted p values are given without correction for microglia proportion and with correction for microglia proportion. Without correction, p values are considered significant at <0.05. With correction, p values are considered significant at <0.10.

| **Gene symbol** | **Gene name** | **MS nodules vs MS nnNAWM** | | | | **Stroke nodules vs stroke nnNAWM** | | | | **MS nodules vs stroke nodules** | | | |
|---|---|---|---|---|---|---|---|---|---|---|---|---|---|
| | | LogFC | P. Adj. | LogFC cor. | P. Adj. Cor. | LogFC | P.Adj. | LogFC cor. | P. Adj. Cor. | LogFC | P. Adj. | LogFC cor. | P. Adj. Cor. |
| ACLY | ATP citrate lyase | 2.920 | 0.500 | 1.636 | 0.931 | -1.410 | 0.880 | -1.758 | 0.905 | 5.600 | **0.048** | 4.711 | 0.332 |
| ACOX1 | acyl-CoA oxidase 1 | 1.990 | 0.663 | 0.983 | 0.966 | -2.840 | 0.751 | -3.127 | 0.819 | 6.090 | **0.019** | 5.421 | 0.210 |
| APBB1IP | amyloid beta precursor protein binding family B member 1 interacting protein | 8.350 | **0.003** | 7.614 | 0.147 | -2.776 | 0.848 | -3.138 | 0.881 | 7.131 | **0.031** | 6.736 | 0.110 |
| APOC1 | apolipoprotein C1 | 4.670 | 0.172 | 3.538 | 0.715 | 8.730 | **0.005** | 8.497 | **0.009** | 0.268 | 0.929 | -0.510 | 0.923 |
| ASPDH | aspartate dehydrogenase domain containing | 5.160 | 0.063 | 4.436 | 0.574 | -0.417 | 0.968 | -0.629 | 0.969 | 6.360 | **0.013** | 5.893 | 0.165 |
| BID | BH3 interacting domain death agonist | 3.994 | 0.267 | 3.312 | 0.772 | 1.425 | 0.858 | 1.200 | 0.932 | 6.642 | **0.013** | 6.210 | 0.120 |
| C1QA | complement C1q A chain | 9.450 | **1.40e-5** | 8.344 | **0.022** | 5.120 | 0.583 | 4.966 | 0.758 | 1.150 | 0.722 | 0.309 | 0.964 |
| C1QB | complement C1q B chain | 8.310 | **0.001** | 7.831 | **0.037** | 9.040 | **0.001** | 8.891 | **0.002** | 1.160 | 0.641 | 0.859 | 0.834 |
| CASP3 | caspase 3 | 5.850 | **0.030** | 5.359 | 0.436 | -1.100 | 0.900 | -1.223 | 0.935 | 6.040 | **0.023** | 5.692 | 0.200 |
| CD14 | CD14 molecule | 7.690 | **0.002** | 6.245 | 0.231 | 2.270 | 0.826 | 1.746 | 0.913 | 5.370 | 0.083 | 4.488 | 0.303 |
| CD83 | CD83 molecule | 7.370 | **0.002** | 4.835 | 0.436 | 4.020 | 0.583 | 3.174 | 0.819 | 2.830 | 0.392 | 1.182 | 0.828 |
| CD84 | CD84 molecule | 8.169 | **0.001** | 5.701 | 0.342 | 3.622 | 0.670 | 2.739 | 0.820 | 4.026 | 0.221 | 2.481 | 0.619 |
| CDKN1A | cyclin dependent kinase inhibitor 1A | 7.150 | **0.004** | 5.255 | 0.404 | 5.960 | 0.202 | 5.364 | 0.417 | -0.766 | 0.843 | -2.051 | 0.700 |
| CHI3L1 | chitinase 3 like 1 | 6.900 | **0.050** | 5.630 | 0.515 | 7.540 | 0.140 | 7.100 | 0.274 | 0.748 | 0.845 | -0.083 | 0.992 |
| CHI3L2 | chitinase 3 like 2 | 7.007 | **0.009** | 4.121 | 0.615 | 0.535 | 0.968 | -0.464 | 0.988 | 3.747 | 0.269 | 1.913 | 0.716 |
| COA4 | cytochrome c oxidase assembly factor 4 homolog | 3.260 | 0.430 | 3.725 | 0.756 | -1.690 | 0.848 | -1.525 | 0.918 | 6.300 | **0.021** | 6.595 | 0.131 |
| CORO1A | coronin 1A | 3.750 | 0.413 | 2.484 | 0.876 | -0.065 | 0.994 | -0.467 | 0.985 | 6.720 | **0.029** | 5.846 | 0.214 |
| CRYZL1 | crystallin zeta like 1 | 2.000 | 0.672 | 1.007 | 0.967 | -0.108 | 0.991 | -0.461 | 0.985 | 5.950 | **0.040** | 5.317 | 0.235 |
| CXCL16 | C-X-C motif chemokine ligand 16 | 10.100 | **2.11e-6** | 9.666 | **0.004** | 2.220 | 0.860 | 2.096 | 0.918 | 4.590 | 0.140 | 4.329 | 0.317 |
| DAGLB | diacylglycerol lipase beta | 7.430 | **0.001** | 7.622 | **0.053** | -0.050 | 0.994 | 0.011 | 1.000 | 6.660 | **0.005** | 6.787 | **0.068** |

| | | | | | | | | | | | | | |
|---|---|---|---|---|---|---|---|---|---|---|---|---|---|
| EGR1 | early growth response 1 | 6.460 | **0.003** | 5.034 | 0.353 | 3.130 | 0.628 | 2.587 | 0.819 | 2.810 | 0.318 | 1.954 | 0.667 |
| FABP5 | fatty acid binding protein 5 | -0.395 | 0.937 | -1.280 | 0.931 | -4.790 | 0.491 | -5.000 | 0.557 | 7.600 | **0.002** | 6.942 | **0.056** |
| FADD | Fas associated via death domain | 5.300 | **0.036** | 3.033 | 0.736 | 0.248 | 0.968 | -0.296 | 0.988 | 4.830 | 0.066 | 3.150 | 0.440 |
| GPCPD1 | glycerophosphocholine phosphodiesterase 1 | 3.100 | 0.439 | 2.102 | 0.887 | -1.470 | 0.858 | -1.770 | 0.894 | 6.050 | **0.024** | 5.369 | 0.226 |
| GPNMB | glycoprotein nmb | 7.200 | **0.013** | 6.042 | 0.321 | 6.620 | 0.185 | 6.224 | 0.322 | 2.670 | 0.425 | 1.893 | 0.704 |
| GRB2 | growth factor receptor bound protein 2 | 5.330 | 0.144 | 4.316 | 0.682 | 2.250 | 0.956 | 0.690 | 0.981 | 6.490 | **0.046** | 5.939 | 0.192 |
| HLA-DMA | major histocompatibility complex, class II, DM alpha | 6.570 | **0.009** | 3.456 | 0.679 | 2.670 | 0.759 | 1.613 | 0.900 | 3.900 | 0.209 | 1.900 | 0.685 |
| HLA-DMB | major histocompatibility complex, class II, DM beta | 5.910 | **0.023** | 3.828 | 0.652 | 1.850 | 0.843 | 1.279 | 0.930 | 3.740 | 0.223 | 2.267 | 0.645 |
| HLA-DPB1 | major histocompatibility complex, class II, DP beta 1 | 7.740 | **0.008** | 7.106 | 0.203 | 3.560 | 0.791 | 3.381 | 0.868 | 3.050 | 0.385 | 2.635 | 0.620 |
| HLA-DRB1 | major histocompatibility complex, class II, DR beta 1 | 6.310 | **0.042** | 5.426 | 0.453 | 4.250 | 0.660 | 3.980 | 0.819 | 4.200 | 0.204 | 3.623 | 0.444 |
| HLA-DRB5 | major histocompatibility complex, class II, DR beta 5 | 6.040 | **0.032** | 6.039 | 0.321 | 0.840 | 0.923 | 0.847 | 0.960 | 7.640 | **0.002** | 7.632 | **0.037** |
| IAH1 | isoamyl acetate hydrolyzing esterase 1 (putative) | 6.480 | **0.024** | 6.752 | 0.196 | -0.833 | 0.960 | -0.737 | 0.978 | 7.050 | **0.008** | 7.222 | **0.056** |
| IDH1 | isocitrate dehydrogenase (NADP(+)) 1 | 3.820 | 0.213 | 2.983 | 0.781 | -1.340 | 0.859 | -1.586 | 0.900 | 5.090 | **0.047** | 4.505 | 0.299 |
| IFI27 | interferon alpha inducible protein 27 | 6.480 | **0.030** | 5.794 | 0.447 | 1.760 | 0.858 | 1.504 | 0.930 | 5.150 | 0.112 | 4.701 | 0.358 |
| IFNAR2 | interferon alpha and beta receptor subunit 2 | 1.050 | 0.851 | 2.011 | 0.894 | 0.015 | 0.997 | 0.305 | 0.989 | 6.190 | **0.016** | 6.851 | **0.085** |
| IL18 | interleukin 18 | 8.770 | **1.21e-5** | 8.771 | **0.008** | 3.320 | 0.633 | 3.306 | 0.786 | 4.940 | 0.070 | 4.949 | 0.206 |
| IL1B | interleukin 1 beta | 6.260 | **0.009** | 5.264 | 0.395 | 1.380 | 0.848 | 1.061 | 0.937 | 4.350 | 0.109 | 3.697 | 0.404 |
| IL33 | interleukin 33 | 5.550 | 0.073 | 5.429 | 0.509 | -0.597 | 0.960 | -0.643 | 0.974 | 6.650 | **0.017** | 6.553 | 0.164 |
| IRF8 | interferon regulatory factor 8 | 5.420 | **0.030** | 3.144 | 0.728 | 2.000 | 0.789 | 1.439 | 0.902 | 2.900 | 0.308 | 1.224 | 0.806 |
| ISG15 | ISG15 ubiquitin like modifier | 3.660 | 0.346 | 2.536 | 0.847 | 0.248 | 0.968 | -0.079 | 1.000 | 7.460 | **0.004** | 6.667 | **0.098** |
| JAK3 | Janus kinase 3 | 5.950 | **0.016** | 4.318 | 0.533 | 0.511 | 0.963 | 0.134 | 0.997 | 4.430 | 0.096 | 3.206 | 0.451 |
| LPL | lipoprotein lipase | 7.920 | **0.002** | 5.355 | 0.395 | 5.300 | 0.436 | 4.687 | 0.611 | 2.390 | 0.515 | 0.489 | 0.951 |
| LRRK2 | leucine rich repeat kinase 2 | 5.940 | **0.033** | 6.018 | 0.351 | 3.350 | 0.685 | 3.376 | 0.819 | 4.550 | 0.140 | 4.614 | 0.326 |
| MAOB | monoamine oxidase B | 1.965 | 0.654 | 0.757 | 0.975 | 0.284 | 0.978 | -0.242 | 0.997 | 6.603 | **0.039** | 5.981 | 0.165 |
| MERTK | MER proto-oncogene, tyrosine kinase | 5.090 | **0.023** | 3.533 | 0.587 | 0.248 | 0.968 | -0.164 | 0.995 | 4.330 | 0.063 | 3.208 | 0.399 |
| MTG1 | mitochondrial ribosome associated GTPase 1 | 6.500 | **0.017** | 8.188 | **0.070** | -0.309 | 0.968 | 0.066 | 1.000 | 5.820 | **0.041** | 7.089 | **0.080** |
| MX1 | MX dynamin like GTPase 1 | 8.930 | **1.40e-5** | 8.283 | **0.022** | 1.330 | 0.870 | 1.072 | 0.939 | 6.420 | **0.013** | 6.033 | 0.101 |
| NCF2 | neutrophil cytosolic factor 2 | 5.820 | **0.033** | 3.204 | 0.743 | 1.720 | 0.830 | 1.094 | 0.937 | 3.590 | 0.224 | 1.637 | 0.747 |
| NCKAP1L | NCK associated protein 1 like | 8.490 | **1.46e-5** | 7.309 | **0.033** | 2.430 | 0.725 | 1.985 | 0.847 | 5.540 | **0.032** | 4.827 | 0.196 |
| PARP9 | poly(ADP-ribose) polymerase family member 9 | 5.970 | 0.098 | 6.246 | 0.445 | -1.180 | 0.920 | -1.330 | 0.947 | 7.280 | **0.013** | 7.503 | **0.080** |

| | | | | | | | | | | | | | |
|---|---|---|---|---|---|---|---|---|---|---|---|---|---|
| PLCD3 | phospholipase C delta 3 | 1.910 | 0.673 | 0.620 | 0.978 | -1.370 | 0.880 | -1.718 | 0.900 | 5.500 | **0.037** | 4.621 | 0.285 |
| PPRC1 | PPARG related coactivator 1 | 5.700 | **0.030** | 6.041 | 0.353 | -1.270 | 0.880 | -1.148 | 0.942 | 5.680 | **0.037** | 5.906 | 0.196 |
| PSME3 | proteasome activator subunit 3 | 3.610 | 0.386 | 2.132 | 0.898 | -0.468 | 0.968 | -0.853 | 0.964 | 6.100 | **0.037** | 5.028 | 0.303 |
| SAMM50 | SAMM50 sorting and assembly machinery component | 5.857 | **0.027** | 3.988 | 0.634 | -0.806 | 0.956 | -1.347 | 0.930 | 5.516 | **0.047** | 4.317 | 0.340 |
| SLC11A1 | solute carrier family 11 member 1 | 7.072 | **0.004** | 5.312 | 0.378 | 8.227 | **0.005** | 7.755 | **0.010** | -1.669 | 0.628 | -2.926 | 0.544 |
| SMAD3 | SMAD family member 3 | 2.670 | 0.529 | 1.611 | 0.930 | 0.547 | 0.960 | 0.268 | 0.993 | 5.660 | **0.037** | 4.883 | 0.285 |
| SPP1 | secreted phosphoprotein 1 | 3.060 | **0.050** | 1.371 | 0.746 | 3.120 | 0.509 | 2.524 | 0.696 | 0.502 | 0.723 | -0.585 | 0.739 |
| STARD13 | STAR related lipid transfer domain containing 13 | 2.490 | 0.569 | 3.656 | 0.744 | -0.422 | 0.968 | -0.073 | 1.000 | 6.620 | **0.013** | 7.404 | **0.068** |
| TCIRG1 | T cell immune regulator 1, ATPase H+ transporting V0 subunit a3 | 6.470 | **0.019** | 4.461 | 0.561 | 0.295 | 0.968 | -0.391 | 0.987 | 4.860 | 0.112 | 3.609 | 0.437 |
| TLR2 | toll like receptor 2 | 5.930 | **0.020** | 4.506 | 0.541 | 0.887 | 0.927 | 0.441 | 0.982 | 3.730 | 0.190 | 2.779 | 0.564 |
| TLR6 | toll like receptor 6 | 6.780 | **0.006** | 5.903 | 0.342 | 1.180 | 0.875 | 0.897 | 0.952 | 5.090 | 0.062 | 4.507 | 0.321 |
| WASL | WASP like actin nucleation promoting factor | 0.444 | 0.941 | 0.719 | 0.976 | 7.780 | **0.018** | 7.885 | **0.033** | -1.750 | 0.542 | -1.580 | 0.752 |

**Supplementary table 8: Significantly enriched GO classes in MS nodules vs MS nnNAWM**. Gene ontology analysis was used to find genes of interest based on function. The column count indicates the number of genes likely associated with the GO term and the unadjusted p value is shown. P-values were obtained by a Modified Fisher Exact one-sided test, calculating the EASE score.

| Term | GO class | Count | P |
|---|---|---|---|
| positive regulation of interferon-gamma production | GO:0032729 | 13 | 1.79e-9 |
| positive regulation of tumor necrosis factor production | GO:0032760 | 13 | 6.65e-8 |
| peptide antigen assembly with MHC class II protein complex | GO:0032755 | 6 | 2.58e-6 |
| positive regulation of interleukin-6 production | GO:0032755 | 11 | 2.93e-6 |
| immunoglobulin production involved in immunoglobulin mediated immune response | GO:0002381 | 6 | 3.61e-6 |
| antigen processing and presentation of exogenous peptide antigen via MHC class II | GO:0019886 | 7 | 4.30e-6 |
| positive regulation of interleukin-8 production | GO:0032757 | 9 | 4.94e-6 |
| antigen processing and presentation of peptide or polysaccharide antigen via MHC class II | GO:0002504 | 6 | 8.71e-6 |
| immune response | GO:0006955 | 22 | 1.43e-5 |
| defense response to virus | GO:0051607 | 14 | 4.11e-5 |
| negative regulation of interferon-gamma production | GO:0032689 | 7 | 4.90e-5 |
| positive regulation of monocyte chemotactic protein-1 production | GO:0071639 | 5 | 7.33e-5 |
| cortical actin cytoskeleton organization | GO:0030866 | 6 | 8.36e-5 |
| erythrocyte differentiation | GO:0030218 | 7 | 1.01e-4 |
| positive regulation of T cell activation | GO:0050870 | 6 | 1.14e-4 |
| innate immune response | GO:0045087 | 22 | 2.22e-4 |
| positive regulation of interleukin-1 beta production | GO:0032731 | 7 | 3.89e-4 |
| positive regulation of T cell proliferation | GO:0042102 | 7 | 3.89e-4 |
| intracellular signal transduction | GO:0035556 | 18 | 3.99e-4 |
| positive regulation of interleukin-12 production | GO:0032735 | 6 | 4.10e-4 |
| inflammatory response | GO:0006954 | 17 | 4.24e-4 |
| positive regulation of NIK/NF-kappaB signaling | GO:1901224 | 7 | 0.001 |
| cellular response to lipopolysaccharide | GO:0071222 | 11 | 0.001 |
| antigen processing and presentation | GO:0019882 | 6 | 0.001 |
| positive regulation of NF-kappaB transcription factor activity | GO:0051092 | 10 | 0.001 |
| receptor internalization | GO:0031623 | 6 | 0.001 |
| cell activation | GO:0001775 | 4 | 0.001 |
| microglial cell activation | GO:0001774 | 5 | 0.001 |

| | | | |
|---|---|---|---|
| T cell homeostasis | GO:0043029 | 5 | 0.001 |
| dendritic cell differentiation | GO:0097028 | 4 | 0.001 |
| response to tumor necrosis factor | GO:0034612 | 5 | 0.002 |
| cellular response to diacyl bacterial lipopeptide | GO:0071726 | 3 | 0.002 |
| positive regulation of I-kappaB kinase/NF-kappaB signaling | GO:0043123 | 10 | 0.002 |
| lipopolysaccharide-mediated signaling pathway | GO:0031663 | 5 | 0.003 |
| positive regulation of interleukin-10 production | GO:0032733 | 5 | 0.003 |
| positive regulation of superoxide anion generation | GO:0032930 | 4 | 0.003 |
| phagocytosis | GO:0006909 | 6 | 0.004 |
| negative regulation of T cell proliferation | GO:0042130 | 5 | 0.005 |
| neutrophil activation involved in immune response | GO:0002283 | 3 | 0.006 |
| positive regulation of T-helper 1 cell cytokine production | GO:2000556 | 3 | 0.006 |
| negative regulation of interleukin-10 production | GO:0032693 | 4 | 0.006 |
| defense response | GO:0006952 | 6 | 0.006 |
| positive regulation of phagocytosis | GO:0050766 | 5 | 0.006 |
| positive regulation of interleukin-17 production | GO:0032740 | 4 | 0.006 |
| signal transduction | GO:0007165 | 31 | 0.007 |
| positive regulation of interleukin-4 production | GO:0032753 | 4 | 0.007 |
| extrinsic apoptotic signaling pathway | GO:0097191 | 5 | 0.007 |
| positive regulation of gene expression | GO:0010628 | 16 | 0.010 |
| substrate adhesion-dependent cell spreading | GO:0034446 | 5 | 0.011 |
| positive regulation of transcription from RNA polymerase II promoter | GO:0045944 | 29 | 0.011 |
| calcium-mediated signaling | GO:0019722 | 6 | 0.012 |
| positive regulation of cell migration | GO:0030335 | 10 | 0.014 |
| extrinsic apoptotic signaling pathway in absence of ligand | GO:0097192 | 4 | 0.015 |
| platelet activation | GO:0030168 | 5 | 0.015 |
| organelle transport along microtubule | GO:0072384 | 3 | 0.018 |
| positive regulation of granulocyte macrophage colony-stimulating factor production | GO:0032725 | 3 | 0.020 |
| negative regulation of activated T cell proliferation | GO:0046007 | 3 | 0.020 |
| regulation of tumor necrosis factor-mediated signaling pathway | GO:0010803 | 3 | 0.023 |
| positive regulation of inflammatory response | GO:0050729 | 6 | 0.024 |
| integrin-mediated signaling pathway | GO:0007229 | 6 | 0.024 |
| positive regulation of neuron death | GO:1901216 | 4 | 0.025 |
| apoptotic signaling pathway | GO:0097190 | 5 | 0.026 |

| | | | |
|---|---|---|---|
| negative regulation of tumor necrosis factor production | GO:0032720 | 5 | 0.027 |
| positive regulation of protein ubiquitination | GO:0031398 | 5 | 0.028 |
| response to lipopolysaccharide | GO:0032496 | 7 | 0.028 |
| cytokine-mediated signaling pathway | GO:0019221 | 7 | 0.028 |
| late endosome to lysosome transport | GO:1902774 | 3 | 0.028 |
| negative regulation of T cell activation | GO:0050868 | 3 | 0.028 |
| positive regulation of nitric-oxide synthase biosynthetic process | GO:0051770 | 3 | 0.028 |
| positive regulation of B cell proliferation | GO:0030890 | 4 | 0.029 |
| positive regulation of chemokine production | GO:0032722 | 4 | 0.029 |
| animal organ regeneration | GO:0031100 | 4 | 0.029 |
| platelet aggregation | GO:0070527 | 4 | 0.029 |
| regulation of small GTPase mediated signal transduction | GO:0051056 | 6 | 0.029 |
| negative regulation of leukocyte apoptotic process | GO:2000107 | 2 | 0.029 |
| toll-like receptor TLR6:TLR2 signaling pathway | GO:0038124 | 2 | 0.029 |
| methotrexate transport | GO:0051958 | 2 | 0.029 |
| detection of diacyl bacterial lipopeptide | GO:0042496 | 2 | 0.029 |
| regulation of G-protein coupled receptor protein signaling pathway | GO:0008277 | 4 | 0.032 |
| angiogenesis | GO:0001525 | 9 | 0.034 |
| positive regulation of nitric oxide biosynthetic process | GO:0045429 | 4 | 0.036 |
| adaptive immune response | GO:0002250 | 13 | 0.036 |
| B cell differentiation | GO:0030183 | 5 | 0.038 |
| MyD88-dependent toll-like receptor signaling pathway | GO:0002755 | 3 | 0.041 |
| stimulatory C-type lectin receptor signaling pathway | GO:0002223 | 3 | 0.041 |
| MAPK cascade | GO:0000165 | 6 | 0.041 |
| negative regulation of lymphocyte activation | GO:0051250 | 2 | 0.044 |
| reduced folate transmembrane transport | GO:0098838 | 2 | 0.044 |
| nitric oxide metabolic process | GO:0046209 | 2 | 0.044 |
| positive regulation of T cell activation via T cell receptor contact with antigen bound to MHC molecule on antigen presenting cell | GO:2001190 | 2 | 0.044 |
| cellular response to triacyl bacterial lipopeptide | GO:0071727 | 2 | 0.044 |
| positive regulation of glial cell proliferation | GO:0060252 | 3 | 0.045 |
| cell projection assembly | GO:0030031 | 3 | 0.045 |
| transmembrane receptor protein tyrosine kinase signaling pathway | GO:0007169 | 6 | 0.046 |
| positive regulation of protein complex assembly | GO:0031334 | 4 | 0.047 |
| toll-like receptor signaling pathway | GO:0002224 | 3 | 0.048 |

**Supplementary table 9: Significantly enriched GO classes in MS nodules vs stroke nodules.** Gene ontology analysis was used to find genes of interest based on function. The column count indicates the number of genes likely associated with the GO term and the unadjusted p value is shown. P-values were obtained by a Modified Fisher Exact one-sided test, calculating the EASE score.

| **Term** | **GO class** | **Count** | **P** |
|---|---|---|---|
| regulation of catalytic activity | GO:0050790 | 13 | 0.001 |
| protein transport | GO:0015031 | 14 | 0.001 |
| Golgi to plasma membrane transport | GO:0006893 | 4 | 0.003 |
| protein targeting to mitochondrion | GO:0006626 | 4 | 0.003 |
| regulation of small GTPase mediated signal transduction | GO:0051056 | 6 | 0.009 |
| membrane fission | GO:0090148 | 4 | 0.009 |
| ER to Golgi vesicle-mediated transport | GO:0006888 | 6 | 0.014 |
| intrinsic apoptotic signaling pathway in response to DNA damage | GO:0008630 | 4 | 0.017 |
| mitochondrial translation | GO:0032543 | 5 | 0.020 |
| regulation of mitochondrial DNA metabolic process | GO:1901858 | 2 | 0.021 |
| actin cytoskeleton reorganization | GO:0031532 | 4 | 0.026 |
| lipid metabolic process | GO:0006629 | 7 | 0.026 |
| Rho protein signal transduction | GO:0007266 | 4 | 0.027 |
| regulation of Rho protein signal transduction | GO:0035023 | 3 | 0.037 |
| response to aluminum ion | GO:0010044 | 2 | 0.043 |
| glial cell apoptotic process | GO:0034349 | 2 | 0.043 |
| T cell homeostasis | GO:0043029 | 3 | 0.047 |
| actin cytoskeleton organization | GO:0030036 | 6 | 0.048 |

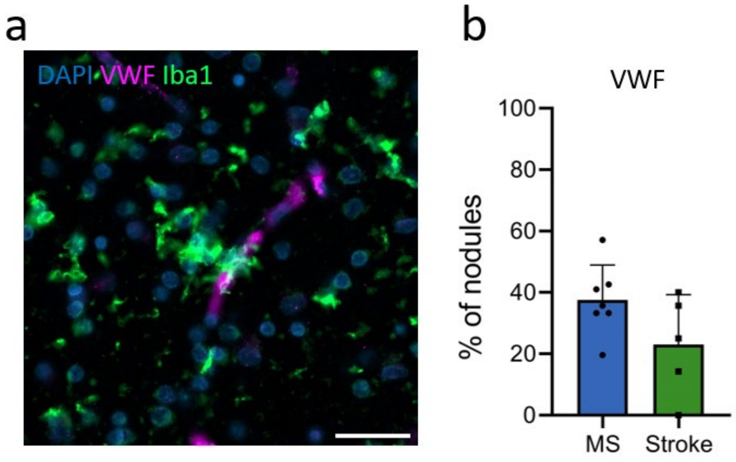


**Supplementary figure 1:** A) Immunohistochemistry staining of iba1$^+$ nodule (in green) in MS in contact with a VWF$^+$ vessel (in magenta). B) Nodules in MS and in stroke are equally often in contact with vessels, quantified in n=7 MS donors and n=5 stroke donors. Data is shown as mean ± standard deviation. Scalebar = 35 µm. Source data are provided as a Source Data file.

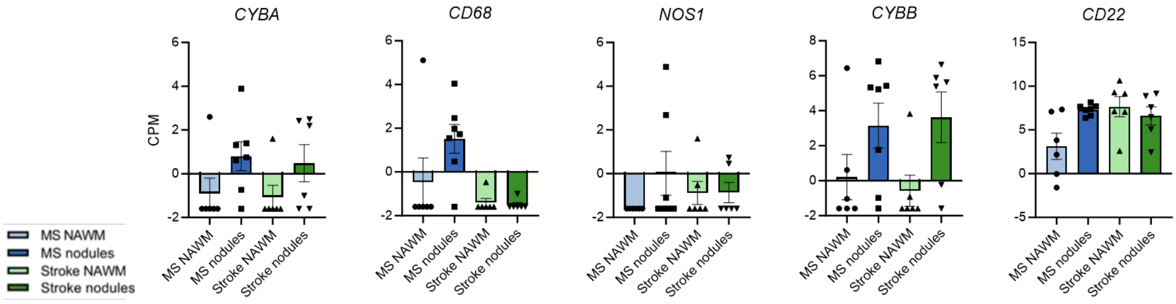


**Supplementary Figure 2:** Phagocytic exhaustion markers are not significantly differentially expressed in MS nodules compared to stroke nodules. Analysis performed on n=6 MS NAWM samples, n=7 MS nodule samples, n=6 stroke NAWM samples, n=6 stroke nodule samples. Data is shown as mean ± standard deviation. Source data are provided as a Source Data file.

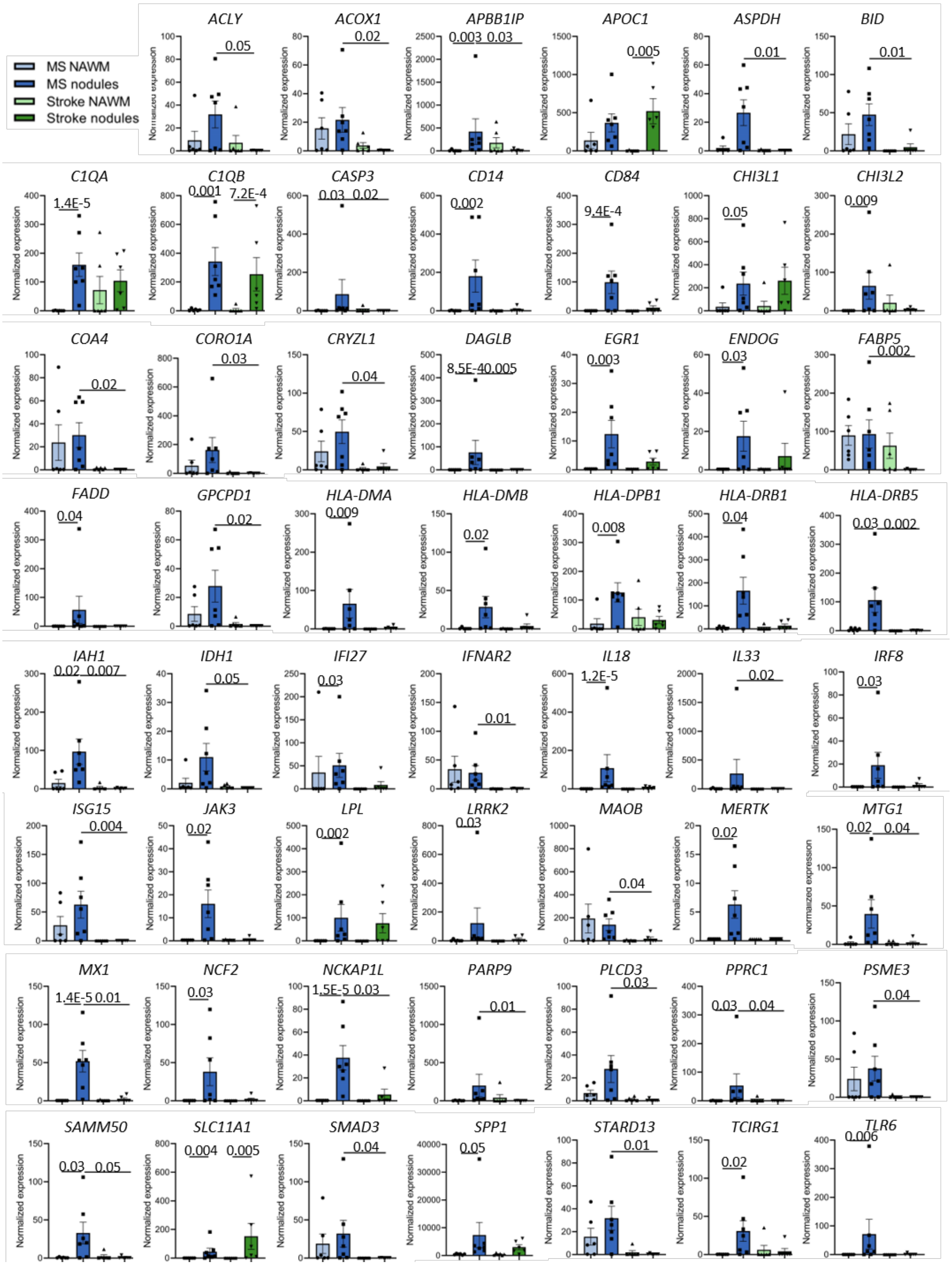


**Supplementary figure 3:** Normalized expression of genes likely involved in functional pathways relevant for MS pathology. Analysis performed on n=6 MS NAWM samples, n=7 MS nodule samples, n=6 stroke NAWM samples, n=6 stroke nodule samples. Data is shown as mean ± standard deviation. Normalized gene expression is calculated as 2log(CPM). p value <0.05 is indicated with *, <0.01 is indicated with **, <0.001 is indicated with ***. Source data are provided as a Source Data file.

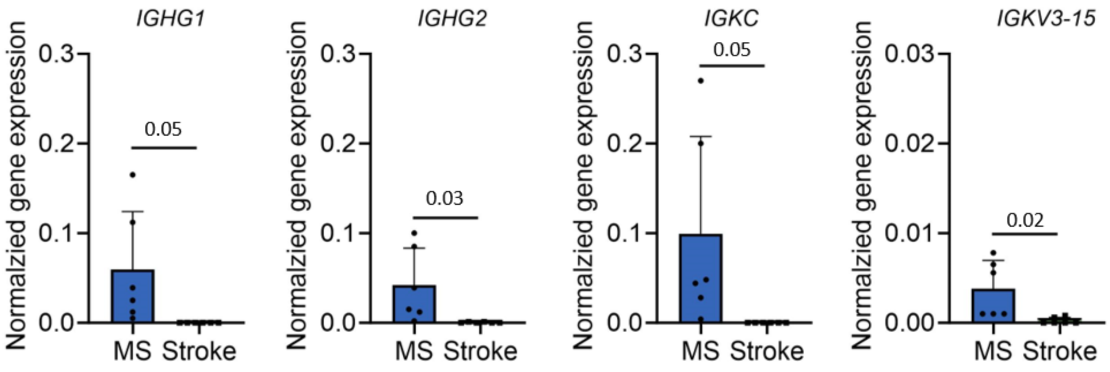


**Supplementary figure 4:** Immunoglobulin gene expression quantified with RT-qPCR of MS (n=6) and stroke (n=6) (NA)WM tissue containing nodules showing higher expression in MS compared to stroke. Data is shown as mean ± standard deviation. Source data are provided as a Source Data file.

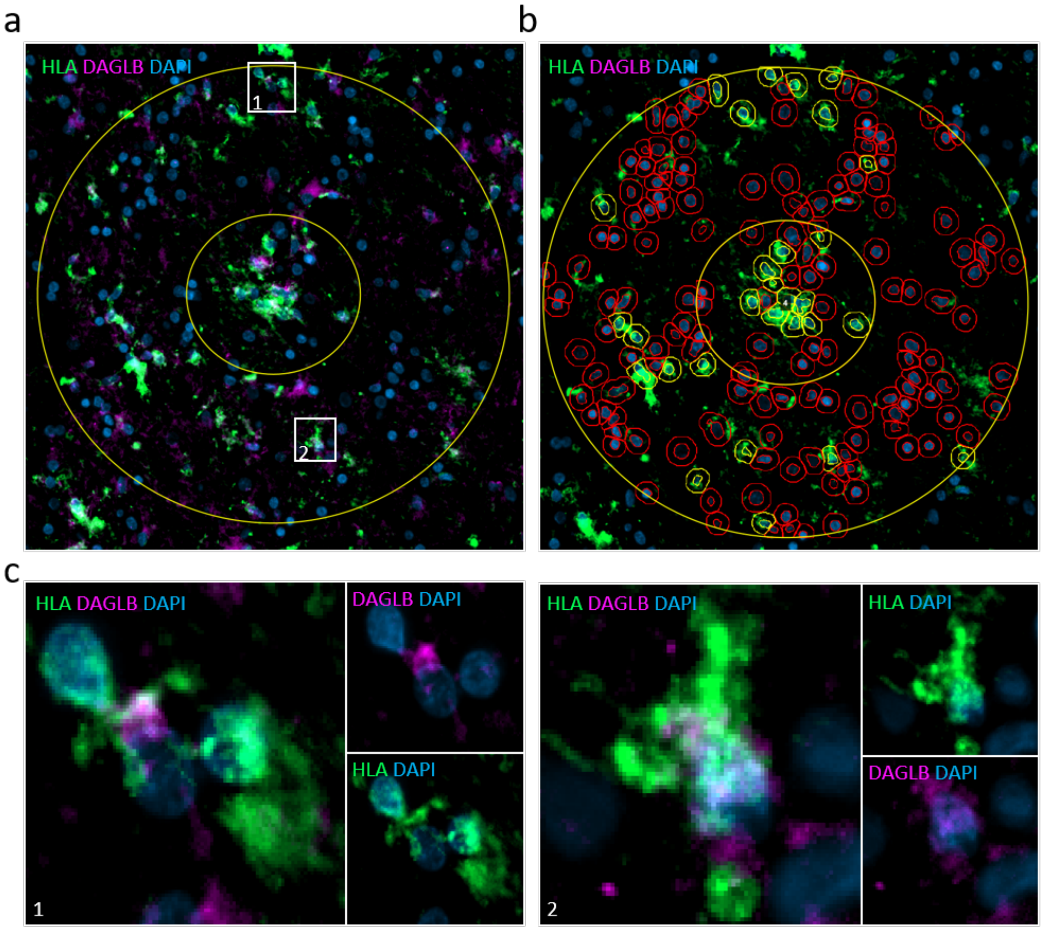


**Supplementary figure 5:** Exemplary figure of quantification of (NA)WM microglia adjacent to microglia nodules for DAGLB, FABP5, IAH1 and STARD13. A) for each nodule, an expansion of 100 µm was created of the annotation. B) Using cell-detection in QuPath, all HLA+ cells were annotated with green, all HLA- cells were annotated with red. Each HLA+ cell was annotated as C) negative (left panel) or positive (right panel) for DAGLB.
